# Identification of *Burkholderia cenocepacia* non-coding RNAs expressed during *Caenorhabditis elegans* infection

**DOI:** 10.1007/s00253-023-12530-3

**Published:** 2023-04-25

**Authors:** Tiago Pita, Joana R. Feliciano, Jorge H. Leitão

**Affiliations:** Department of Bioengineering, iBB-Institute for Bioengineering and Biosciences, and Associate Laboratory i4HB-Institute for Health and Bioeconomy at Instituto Superior Técnico, Instituto Superior Técnico, Universidade de Lisboa, 1049-001 Lisbon, Portugal

**Keywords:** *Burkholderia cenocepacia*, *C. elegans* infection model, RNA-seq, Non-coding RNAs

## Abstract

**Abstract:**

Small non-coding RNAs (sRNAs) are key regulators of post-transcriptional gene expression in bacteria. Despite the identification of hundreds of bacterial sRNAs, their roles on bacterial physiology and virulence remain largely unknown, as is the case of bacteria of the *Burkholderia cepacia* complex (Bcc). Bcc is a group of opportunistic pathogens with relatively large genomes that can cause lethal lung infections amongst cystic fibrosis (CF) patients. To characterise sRNAs expressed by Bcc bacteria when infecting a host, the nematode *Caenorhabditis elegans* was used as an infection model by the epidemic CF strain *B. cenocepacia* J2315. A total of 108 new and 31 previously described sRNAs with a predicted Rho independent terminator were identified, most of them located on chromosome 1. RIT11b, a sRNA downregulated under *C. elegans* infection conditions, was shown to directly affect *B. cenocepacia* virulence, biofilm formation, and swimming motility. RIT11b overexpression reduced the expression of the direct targets *dusA* and *pyrC*, involved in biofilm formation, epithelial cell adherence, and chronic infections in other organisms. The in vitro direct interaction of RIT11b with the *dusA* and *pyrC* messengers was demonstrated by electrophoretic mobility shift assays. To the best of our knowledge this is the first report on the functional characterization of a sRNA directly involved in *B. cenocepacia* virulence.

**Key points:**

*• 139 sRNAs expressed by B. cenocepacia during C. elegans infection were identified*

*• The sRNA RIT11b affects B. cenocepacia virulence, biofilm formation, and motility*

*• RIT11b directly binds to and regulates dusA and pyrC mRNAs*

**Supplementary Information:**

The online version contains supplementary material available at 10.1007/s00253-023-12530-3.

## Introduction

The *Burkhoderia cepacia* complex (Bcc) comprises at least 24 Gram-negative *Betaproteobacteria* species (Martina et al. 2018), all of them able to colonise and infect immunocompromised individuals and cystic fibrosis (CF) patients. The clinical outcome of Bcc infections is highly variable and strain-dependent, in some cases leading to a clinical condition of lethal uncontrolled deterioration with septicaemia and necrotising pneumonia, known as the cepacia syndrome (Golshahi et al. 2011; Leitão et al. 2017). Bcc strains are intrinsically resistant to most antibiotics (Rhodes and Schweizer 2016), turning those infections difficult to eradicate. *Burkholderia cenocepacia* and *Burkholderia multivorans* are the most predominant Bcc species infecting CF patients worldwide (Hassan et al. 2020). *B. cenocepacia* possess a large genome (approximately 8 Mb), composed by multiple replicons, the chromosome 1 (3.9 Mb), chromosome 2 (3.2 Mb), megaplasmid pC3 (0.88 Mb), and plasmid pBCJ2315 (92 kb) (Holden et al. 2009). At least 7261 coding sequences, six sets of rRNA genes, and 74 tRNAs are annotated in the genome of *B. cenocepacia* J2315, the representative member of the epidemic ET12 lineage that was isolated from a CF patient (Holden et al. 2009). Other regulatory non-coding sequences, like small non-coding RNAs (sRNAs), are also present within the *B. cenocepacia* genome; however, these RNA molecules remain poorly characterised and their study in the context of infection is almost non-existent (Pita et al. 2018).

Small non-coding RNAs (sRNAs) emerged as major regulators of physiological processes in bacteria and play important roles on their virulence. The post-transcriptional regulation mediated by sRNAs is particularly important for bacterial pathogens, since these RNA molecules can modulate their target gene expression allowing a fast response towards an efficient adaptation to a challenging and dynamic host environment. The stability and function of these regulatory RNAs often require RNA chaperones, like the proteins Hfq and ProQ (Feliciano et al. 2020; Djapgne and Oglesby 2021). *Pseudomonas aeruginosa*, an organism that is also responsible for severe infections amongst CF patients, possesses several characterised sRNAs involved in regulating key mechanisms for its virulence and pathogenicity, including quorum sensing, iron homeostasis, biofilm formation, response to stress, and host cell invasion (Pusic et al. 2021). In *B. cenocepacia*, 167 putative sRNAs were identified under conditions like growth in CF sputum medium, biofilm formation, and oxidative stress, and very few have been functionally characterised so far (Pita et al. 2018). These include the ncS35 sRNA, which attenuates the growth and reduces the metabolic rate of *B. cenocepacia* J2315 (Kiekens et al. 2018); the ncS27 sRNA that can regulate the metabolic shutdown upon nutrient deprivation (Sass et al. 2019); and BrrF that acts as a regulator of central metabolism and oxidative stress response under iron starvation conditions (Sass and Coenye 2020). Despite the deadly threat of Bcc infections amongst immunocompromised and CF patients, the virulence and unpredictable outcome of Bcc infections remain uncertain and the contribution of sRNAs to Bcc virulence is unknown.

The well-studied organism *Caenorhabditis elegans* has been widely used as a model to study host–pathogen interactions. This animal is genetically well characterised and represents a good model for research due to its experimental simplicity, low cost, and easy handling without raising major ethical concerns (Sánchez-Diener et al. 2017). Furthermore, at least 83% of the *C. elegans* proteome has human homologous genes (Lai et al. 2000), and several studies have been carried out on *C. elegans* to unveil some features of CF pathogens, like *P. aeruginosa* and Bcc bacteria. Some Bcc bacteria have been proven to be virulent to *C. elegans* (Cooper et al. 2009), and a list of genes required for *B. cenocepacia* survival within the host-associated environment has been already identified (Wong et al. 2018). More recently, sRNAs have been shown to be involved in *C. elegans* avoidance behaviour of *P. aeruginosa* (Kaletsky et al. 2020), evidence that highlights the wide role of sRNAs on host-microbe interaction. *C. elegans* was also used to decipher the *Vibrio parahaemolyticus* virulome, and 19 trans-encoded sRNAs were found to be differentially expressed under infection conditions (Zhang et al. 2020). The most advanced techniques of RNA sequencing have allowed the identification and accurate gene quantification of the entire transcriptome for samples with low inputs of RNA, enabling the bacterial transcriptome analysis under infection conditions (Marsh et al. 2017; Hassan et al. 2019).

In the present work, an experimental methodology was developed, involving the recovery of total RNA from *B. cenocepacia* J2315 infecting *C. elegans*, followed by RNA sequencing, to identify the *B. cenocepacia* J2315 sRNAs expressed under infection conditions. Amongst the 108 new and 31 previously described sRNAs, the RIT11b sRNA was functionally characterised in this work. Results show that this regulatory RNA plays a role in *B. cenocepacia* virulence in *C. elegans*, affecting biofilm formation and swimming motility.

## Materials and methods

### Bacterial strains, plasmids, and culture conditions

Bacterial strains and plasmids used in this study are described in Table 1. When in use, *B. cenocepacia* and *Escherichia coli* strains were maintained on Lennox broth (LB; containing in g·L^−1^, tryptone 10, yeast extract 5, NaCl 5) agar plates, supplemented with 200 μg·mL^−1^ chloramphenicol (*B. cenocepacia*), 25 μg·mL^−1^ chloramphenicol, or 150 μg·mL^−1^ ampicillin (*E. coli*), when appropriate. Unless otherwise stated, liquid cultures were carried out in LB liquid medium supplemented with the appropriate antibiotics with orbital agitation (250 rpm), at 37 °C. Bacterial growth was assessed by measuring the cultures optical density at 640 nm (OD_640_).

**Table 1:** Organism strains and plasmids used in this work

Organism or plasmid	Description	Reference or source
*C. elegans* strains		
*C. elegans* Bristol N2	*C. elegans* wild isolate	Caenorhabditis Genetics Center (CGC)^a^
*C. elegans* DH26	*C. elegans* with a temperature-sensitive mutation, rrf-3(b26) II, rendering worms sterile at 25 °C	Caenorhabditis Genetics Center (CGC)^a^
Bacterial strains		
*B. cenocepacia* J2315 (LMG16656)	Cystic fibrosis clinical isolate (Edinburgh, UK); ET12 lineage reference strain	Govan et al. (1993)
*B. cenocepacia* K56-2 (LMG18863)	Cystic fibrosis clinical isolate (Toronto, Canada); ET12 lineage	Darling et al. (1998)
*Escherichia coli* DH5α	F^−^ φ80*lac*ZΔM15Δ (*lac*ZYA*arg*F) U169 *rec*A1 *end*A1 *hsd*R17 (r_K_^−^, m_K_^+^) *pho*A *sup*E44 λ^−^*thi*-1 *gyr*A96 *rel*A1	Invitrogen (Waltham, MA, USA)
*E. coli* BL21 (DE3)	F^−^ *ompT hsdSB* (r_B_^−^m_B_^−^) *dcm gal* λ(DE3)	Stratagene (La Jolla, CA, USA)
*E. coli* OP50	*C. elegans* feeding strain	Caenorhabditis Genetics Center (CGC)^a^
Plasmids		
pUC19	Cloning vector; high copy number; *lac* promoter; AmpR; multiple cloning site located within the *lacZ* gene	Yanisch-Perron et al. (1985)
pIN29	*ori*_pBBR_ Δ*mob*, Cm^r^, DSRed	Vergunst et al. (2010)
pTAP3	pIN29 overexpressing RIT11b under control of *tac* promoter	This work
pTAP4	pIN29 overexpressing RIT32 under control of *tac* promoter	This work
pTAP5	pIN29 overexpressing RIT55 under control of *tac* promoter	This work
pTAP9	pIN29 containing the antisense sequence for RIT2a silencing	This work
pTAP10	pIN29 containing the antisense sequence for RIT98 silencing	This work
pTAP11	pIN29 containing the antisense sequence for RIT43 silencing	This work

^a^The Caenorhabditis Genetics Center (CGC), University of Minnesota, https://cgc.umn.edu/

### Plasmids

Plasmids and primers used in this work are listed in Table 1 and Supplemental Table S1, respectively. To silence the RIT2a, RIT43, and RIT98 sRNAs, a fully complementarity antisense strategy was used, cloning an antisense sequence of each sRNA (38 to 40 bp) in the pIN29 plasmid under control of the *tac* promoter. The two single-stranded oligonucleotides with complementary sequences (RIT2asilencFW and RIT2asilencRV; RIT98silencFW and RIT98silencRV; RIT43silencFW and RIT43silencRV) were annealed to form a double-strand DNA sequence. For this, each oligonucleotide was diluted to a final concentration of 100 µM using the annealing buffer (10 mM Tris, pH 8.0; 50 mM NaCl; 1 mM ethylenediaminetetraacetic acid [EDTA]). Equal volumes of the equimolar oligonucleotides were mixed and incubated at 95 °C for 5 min to break all the hydrogen bonds. The reaction mixture was allowed to cool slowly to room temperature (< 60 min). The double-strand sequences were inserted into the pUC19 cloning vector previously linearised with the *Hin*cII restriction enzyme (Thermo Scientific, Waltham, MA, USA). After sequencing, each plasmid was digested with *Nde*I/*Xba*I, and the resulting fragments were inserted into the pIN29 plasmid yielding the pTAP9, pTAP10, and pTAP11 plasmids. Plasmids pTAP9, pTAP10, and pTAP11 were used to silence, respectively, RIT2a, RIT98, and RIT43.

The overexpression of RIT11b, RIT32, and RIT55 sRNAs was achieved by cloning the entire sequence of each sRNA into the pIN29 plasmid and expressing it under the control of the *tac* promoter. For this purpose, nested PCRs were performed using the outside primers RIT11bNestFW and RIT11bNestRV, RIT32NestFW and RIT32NestRV, or RIT55NestFW and RIT55NestRV, for the first round of amplifications, and, respectively, the inner primers RIT11bSupFW and RIT11bSupRV, RIT32SupFW and RIT32SupRV, or RIT55SupFW and RIT55SupRV, for the second amplification. The resulting 411-bp amplicon containing RIT11b sequence, the 118-bp fragment containing RIT32, and the 223-bp fragment containing RIT55 were digested with *Nde*I/*Xba*I and cloned into the same restriction sites of the pIN29 vector, yielding the pTAP3, pTAP4, and pTAP5 plasmids, respectively. Plasmids pTAP3, pTAP4, and pTAP5 were used to overexpress, respectively, RIT11b, RIT32, and RIT55.

### Biofilm formation assay

The biofilm formation ability by *B. cenocepacia* was assessed based on the crystal violet assay method previously described (Cunha et al. 2004). Briefly, 96-well polystyrene microtitre plates (Greiner Bio-One, Kremsmünster, Austria) containing LB liquid medium were inoculated with mid-exponential phase bacterial cultures using an initial OD_640_ of 0.05 and incubated at 37 °C for 24 or 48 h without agitation. After gentle washing the wells three times, the adherent cells were stained with a crystal violet solution (1% w/v) for 15 min before a new washing step. Biofilm quantification was performed after dissolving bound crystal violet in 95% ethanol and measuring the absorbance at 590 nm in the microplate reader SpectroStar Nano (BMG Labtech, Ortenberg, Germany). Results are mean values ± SD of at least 12 replicates from 3 independent experiments. Changes in biofilm formation were analysed by two-way ANOVA with a Sidak’s multiple-comparison test using GraphPad Prism version 6.0 for Windows (GraphPad Software, San Diego, CA, USA, www.graphpad.com).

### Swimming motility assays

Swimming motility assays were performed according to methods previously described (Déziel et al. 2001). Briefly, swimming agar plates (1% [w/v] tryptone, 0.5% [w/v] NaCl, 0.3% [w/v] agar) were spot inoculated with 1.5 μL of an overnight bacterial culture at the stationary phase, adequately diluted to obtain an OD_640_ of 2.0. Plates were incubated for a period of 72 h at 37 °C, and the halo diameters were measured at 24-h intervals. Results are mean values ± SD of 3 independent assays, using 5 plates per experiment and per strain. Changes in swimming motility formation were analysed by two-way ANOVA with a Tukey’s multiple-comparison test using GraphPad Prism version 6.0 for Windows (GraphPad Software, San Diego, CA, USA, www.graphpad.com).

### *C. elegans* maintenance and synchronization

*C. elegans* strains Bristol N2 (wild-type) and DH26 (a mutant carrying a temperature-sensitive spermatogenesis mutation) were obtained from the Caenorhabditis Genetics Center (University of Minnesota, Minneapolis, USA) (Brenner 1974). Both strains were maintained at 20 °C on Nematode Growth Medium I plates (NGM I: 1.7% [w/v] agar, 50 mM NaCl, 0.35% [w/v] tryptone, 25 mM KH_2_PO_4_, 1 mM CaCl_2_, 1 mM MgSO_4_, 5 μg·mL^−1^ cholesterol, 2 μg·mL^−1^ uracil, and 50 μg·mL^−1^ of the antifungal nystatin), with the *E. coli* strain OP50 as a food source, and manipulated using established techniques (Hope 1999).

*C. elegans* cultures were synchronised using a well-established procedure (Hope 1999). Briefly, adult worms and eggs were washed off from an NGMI plate with sterile water, and one third of the volume of a sodium hypochlorite (4% [w/v]) and sodium hydroxide (1.6 M) solution was added. The mixture was vigorously vortexed for 5 to 7 min to isolate the hypochlorite-resistant eggs, and after two washing steps with sterile water, the pellet was resuspended in M9 buffer and transferred to NGM I plates seeded with *E. coli* OP50. The plates were incubated at 20 °C to allow the growth of the nematodes until the L4 larval stage.

### Lethality assays

To assess the relative virulence of *B. cenocepacia* K56-2 overexpressing sRNAs RIT11b, RIT32, or RIT55, or silencing sRNAs RIT2a, RIT43, or RIT98, nematode slow-killing assays were performed based on the methods described by Cardona et al. (2005), using the *C. elegans* mutant strain DH26. For this purpose, 50 μL of *B. cenocepacia* cultures at the stationary phase, grown in LB liquid medium during 16 h at 37 °C with agitation (250 rpm), was spread onto the surface of 35-mm-diameter Petri plates containing 4 mL of NGM II agar (1.7% [w/v] agar, 50 mM NaCl, 0.25% peptone, 25 mM KH_2_PO_4_, 1 mM CaCl_2_, 1 mM MgSO_4_, 5 μg·mL^−1^ cholesterol, 2 μg·mL^−1^ uracil, and 50 μg·mL^−1^ of the antifungal nystatin) and incubated for 24 h at 37 °C to achieve the formation of a bacterial lawn. Approximately 20–30 hypochlorite-synchronised L4 larvae of *C. elegans* DH26 were inoculated per plate and incubated at 25 °C. The morphological appearance and the number of live worms were checked each 24 h for 3 days. Worms were considered dead when they failed to respond to touch.

### *C. elegans* colonization and intestinal bacteria recovery for bacterial RNA-seq

*C. elegans* Bristol N2 was used as the animal model to assess the transcriptome of *B. cenocepacia* J2315 under conditions mimicking a persistent infection. For that, 100 μL of overnight culture of *B. cenocepacia* J2315, grown in LB liquid medium at 37 °C with agitation (250 rpm), was spread onto the surface of 100-mm-diameter Petri plates containing 22 mL of NGM II agar and incubated for 24 h at 37 °C to achieve the formation of a bacterial lawn. Hypochlorite-synchronised *C. elegans* Bristol N2 larvae were used and maintained in plates with heat-inactivated *E. coli* (80 °C for 30 min) to avoid contamination of the posterior RNA samples with *E. coli* nucleic acids. No colony forming units (CFUs) were obtained after plating heat-treated *E. coli* suspensions. Once the larvae reached the L3 stage, M9 buffer was used to collect them and 25,000 nematodes were inoculated per plate seeded with *B. cenocepacia* and incubated at 25 °C. A total of 12 plates were used, totalising 300,000 nematodes. After 48 h of infection, the nematodes were collected with M9 buffer, double filtrated with nylon net filters (Merck Millipore, Burlington, MA, USA), and washed 2 times with M9 buffer. The 41-µm pore filter was used to retain the large external bacterial agglomerates whereas the 10-µm pore filter was used to collect the infected *C. elegans* and eliminate the external free bacteria. The nematodes were directly and promptly collected from the filter with TRIzol™ Reagent (Invitrogen, Waltham, MA, USA) and vortexed vigorously until complete homogenization was achieved. *B. cenocepacia* J2315 cells plated on NGMII agar plates as previously described, but in the absence of the nematodes, were used as control.

### Colony forming unit quantification

To determine the *B. cenocepacia* CFUs present in infected *C. elegans*, the nematodes were collected by filtration after 48 h of infection, as previously described. The nematodes were directly collected from the filter using 1 mL M9 buffer and were quantified by direct counting using a stereomicroscope. The same volume was collected to determine the number of external bacteria (unprocessed samples) and the total bacteria associated with the infected nematodes (present both outside and inside the worms). To count the external bacteria, the collected volume was only homogenised, whilst to estimate the total number of bacteria approximately 400 mg of 1.0-mm silicon carbide particles (Biospec, Bartlesville, OK, USA) was added to the samples and vigorously vortexed for 10 min. The resulting suspensions were diluted and plated onto LB media for CFU quantification. CFUs inside *C. elegans* were calculated by subtracting the external CFUs to the CFUs of *C. elegans* lysed by bead samples (total CFUs). CFUs were expressed as CFUs/*C. elegans*.

### RNA extraction and sequencing

Total RNA extraction was performed with the reagent TRIzol™ following the manufacturer’s instructions. After extraction, the RNA samples were sent to the company Vertis Biotechnologie AG (Freising, Germany), where a ribosomal RNA extraction was performed using the Ribo-Zero rRNA Removal Kit (Illumina®, San Diego, CA, USA) using a 1:1 ratio of probes from the Human/Mouse/Rat and Bacteria module. The bacterial RNA enrichment was achieved using the Cappable-seq for prokaryotic transcription start site (TSS) determination (New England Biolabs® Inc., Ipswich, MA, USA). This method consists of capping the 5′ triphosphorylated RNA with 3′-desthiobiotin-TEG-guanosine 5′ triphosphate (DTBGTP) (New England Biolabs® Inc., Ipswich, MA, USA) using the vaccinia capping enzyme (VCE) (New England Biolabs® Inc., Ipswich, MA, USA) for reversible binding of biotinylated RNA species to streptavidin. Using this approach, the 5′ fragment of the primary transcripts was obtained.

The Cappable-seq enriched RNAs were first poly(A)-tailed using poly(A) polymerase. Then, the 5′PPP structures were converted into 5′ monophosphate ends using CapClip acid pyrophosphatase (Cellscript, Madison, WI, USA). Afterwards, an RNA adapter was ligated to the 5′-monophosphate of the RNA. First-strand cDNA synthesis was performed using an oligo(dT)-adapter primer and the M-MLV reverse transcriptase. The resulting cDNAs were PCR-amplified to about 10–20 ng/μL using a high-fidelity DNA polymerase. PCR cycles performed and barcode sequences, which are part of the 5′ and 3′ sequencing adapters, are included in Supplemental Table S2. The cDNAs were purified using the Agencourt AMPure XP kit (Beckman Coulter Genomics, Indianapolis, IN, USA) and analysed by capillary electrophoresis. For Illumina NextSeq sequencing, the cDNA libraries were pooled in approximately equimolar amounts. The cDNA pool was size fractionated in the size range of 200–550 bp using a preparative agarose gel. The 3′ and 5′ adapters and the primers used for PCR amplification were designed for TruSeq sequencing according to the instructions of Illumina (Supplemental Table S2). The cDNA pool was single-read sequenced on an Illumina NextSeq 500 system using 75-bp read length. The Array Express accession number for Cappable-seq data is E-MTAB-12592. Principal component analysis and the heatmap were performed with the ClustVis web tool (https://biit.cs.ut.ee/clustvis/), using the default parameters.

### Transcription start site annotation and classification

The NCBI genome database (Sayers et al. 2021) and the *Burkholderia* genome database (Winsor et al. 2008) were used for *B. cenocepacia* J2315 genome visualization and annotation. Cappable-seq data was visualised with the Integrated Genome Viewer version 2.1.1.8 (Thorvaldsdóttir et al. 2013). Mapping output from Cappable-seq was split by replicon and converted to BAM files using SAMtools 1.14 (Li et al. 2009). BEDtools 2.25.0 (Quinlan and Hall 2010) was also used on TSS calling. TSS calling and clustering were performed using the data obtained from controls and infection samples using a previously developed programme on Linux system (Ettwiller et al. 2016). TSS clustering was performed at a neighbourhood of 10 reads. A TSS was considered when, in at least 2 samples, RRSio > 2. RRSio, the relative read score that reflects the strength of each TSS (Ettwiller et al. 2016), was the normalization adopted in this data analysis. Matching the TSSs with *B. cenocepacia* J2315 genome annotation allowed the categorization of the TSSs as gene TSS (TSS located up to 100 nts upstream of a gene), 5′ UTR TSS (TSS located from 100 to 300 nts upstream of a gene), or intergenic TSS (TSS located over 300 nts from a gene). sRNA identification was performed by matching the intergenic TSSs with the rho-independent terminators obtained by TransTerm v2.08, using the default parameters (Kingsford et al. 2007).

### Differential expression analysis

Differential expression analysis was performed identifying the genes whose TSS normalised read counting was higher than fold change (log2) of 2 between the controls and samples. The KEGG (https://www.genome.jp/kegg/kegg1.html) enrichment analysis was performed using the KOBAS software (Bu et al. 2021) by entering the UniProtKB accession number of all the differently expressed genes, and using the default parameters.

### Target prediction and statistical analysis

TargetRNA2 (Kery et al. 2014) and CopraRNA (Wright et al. 2014) were used for sRNA target prediction (using the default parameters, except for the target input parameters that were defined from 300 nucleotides [nts] upstream to 100 nts downstream of the mRNA translation start site), whilst IntaRNA 2.0 (Mann et al. 2017) was used for an accurate prediction of interactions between a sRNA and a predicted target. NCBI Blastn (Sayers et al. 2021) was used to evaluate sRNA conservation. The statistical analysis of raw RNA-seq data results was performed using the ClustVis web tool with default function parameters (Metsalu and Vilo 2015). GraphPad Prism version 6.0 for Windows (GraphPad Software, San Diego, CA, USA, www.graphpad.com) was used for data statistical analysis and graph design.

### Quantitative RT-PCR

For quantitative RT-PCR, RNA was extracted using the Quick-RNA™ Miniprep Kit (Zymo Research, Irvine, CA, USA) following the manufacturer’s instructions. An additional step of DNAse I treatment was included after the total RNA purification. Extracted RNA was analysed in a 1% agarose TBE (tris-2-amino-2-hydroxymethyl-propane-1,3-diol/borate/EDTA; 10 × TBE contains, in g·L^−1^, 121 Tris-base, 61.8 boric acid, and 7.4 EDTA) gel to assess its quality and the concentration was quantified in NanoDrop One (Thermo Scientific, Waltham, MA, USA) spectrophotometer. cDNA was synthesised from 2 μg RNA using a NZY Reverse Transcriptase (NZYTech, Lisbon, Portugal) and random hexamers (NZYTech, Lisbon, Portugal), following the protocol established by the manufacturer. Quantitative RT-PCR (qPCR) was performed in an Applied Biosystems QuantStudio 5 Real-Time PCR System, using the NZYSpeedy qPCR Green Master Mix (a 2 × ready-to-use reaction mixture containing all components necessary for real-time PCR, including a green intercalating dye, dNTPs, stabilisers, and enhancers, as per manufacturer description) from NZYTech (Lisbon, Portugal), ROX (NZYTech, Lisbon, Portugal). Each reaction contained 100 ng of cDNA, 400 nM of each specific primer (Supplemental Table S1), and the NZYSpeedy qPCR Green Master Mix. A calibration curve was performed for each pair of primers and every sample was run in two technical replicates. Relative expression was calculated with the 2^−ΔΔCT^ method (Livak and Schmittgen 2001) using the 5S rRNA for normalization. Changes in expression were analysed by one-way ANOVA with a Tukey’s multiple-comparison test using GraphPad Prism version 6.0 for Windows (GraphPad Software, San Diego, CA, USA, www.graphpad.com).

### In vitro transcription and electrophoretic mobility shift assay

The DNA fragments used as templates for in vitro transcription of the RIT11b sRNA, BCAL1625 (nucleotides − 39 to 89), and BCAL3351 (nucleotides − 53 to 107) RNAs were amplified from *B. cenocepacia* genome using forward primers containing the T7 promoter at the 5′ end (Supplemental Table S1). RNA sequences were transcribed in vitro using the MEGAscript T7 kit (Ambion, Waltham, MA, USA) with native UTP for RIT11b, or native UTP and Biotin-16-UTP (Roche, Basel, Switzerland) (2:1) for BCAL1625 and BCAL3351 RNAs, followed by DNase I digestion (1 unit) for 30 min at 37 °C. The RNA synthesised was purified on a denaturing gel. For that, the RNA was separated on a denaturing 6% polyacrylamide gel (1 × TBE, 7 M urea), stained with ethidium bromide, and visualised by UV shadowing. The area of the gel expected to contain the band of RNA was cut and soaked in elution buffer (0.1 M sodium acetate, 10 mM EDTA, and 0.1% SDS) overnight with rocking at 8 °C. The RNA was precipitated with 0.1 volume of sodium acetate 3 M, pH 5.2 and 3 volume ethanol, and resuspended in RNAse-free water.

The interactions between the unlabelled sRNA RIT11b and the labelled BCAL1625 or BCAL3351 RNAs were assessed using mobility shift assays with increasing amounts of the unlabelled sRNA and a fixed concentration of the labelled RNAs (0.04 pmol). Labelled RNAs, previously denatured for 1 min at 95 °C and cooled for 5 min on ice, were mixed in a total volume of 10 µL with 1 µg yeast tRNA (Ambion, Waltham, MA, USA) and increasing amounts of unlabelled sRNA in structure buffer (10 mM Tris‐HCl pH 7.5, 100 mM KCl, 10 mM MgCl_2_). The reaction mixtures were incubated at 37 °C for 30 min and mixed with 2 µL of native loading dye (50% [v/v] glycerol, 0.25% [w/v] bromophenol blue, 0.25% [w/v] xylene cyanol FF) immediately before loading on 6% polyacrylamide, 0.5 × TBE (diluted from 10 × TBE) gels. After electrophoresis on 6% polyacrylamide (250 V, 4 °C), samples containing biotin-labelled RNA were electroblotted to Amersham Hybond™-N^+^ positively charged nylon transfer membranes (GE Healthcare, Chicago, IL, USA) using a transBlot® SD (Bio-Rad, Hercules, CA, USA) device at 15 V and 220 mA for 50 min. Labelled RNA was detected by chemiluminescence using a Pierce Chemiluminescent Nucleic Acid Detection Module kit (Thermo Scientific, Waltham, MA, USA) according to the manufacturer’s instructions.

## Results

### Development of a model for bacterial transcriptional analysis using* C. elegans *infected by* B. cenocepacia *J2315 

In our attempt to investigate the *B. cenocepacia* non-coding RNA repertoire expressed when infecting the host, the nematode *C. elegans* was used and infected by *B. cenocepacia* J2315, and a protocol to analyse the bacterial transcriptome during infection was developed. Important bottlenecks had to be overcome, including the large amount of *C. elegans* required for infection, the elimination of the external bacteria collected with the nematodes, and the speed of sample collection to avoid transcriptional adaptation. In brief, after egg preparation via culture synchronization, recovered eggs were seeded in NGMI plates with heat-killed *E. coli* to guarantee that the worms do not get their gut colonised by *E. coli*. The infection process started when nematodes in L3 larval stage were harvested and poured on plates containing previously grown *B. cenocepacia* J2315. The use of a 10-µm pore size filter to collect the infected *C. elegans* failed to discard all external bacteria, present both in a free-living form and as clusters attached to the nematodes. Therefore, an extra filtration step by a 41-µm pore size filter was added to the process, allowing the passage of the nematodes through the filter and the retention of the bacterial clusters. In the established methodology the 48-h infected *C. elegans* were submitted to the described double filtration, first using 41-µm pore size filter to discard bacterial clusters, and then using a 10-µm pore size filter to retain the nematodes and separate them from free bacteria. When collected, *C. elegans* were, on average, infected with 6 × 10^3^ CFUs/worm, a value in accordance with literature (Palominos and Calixto 2020). About 300,000 nematodes were used per replicate, to obtain a total of ~ 10^9^ CFUs per sample. After filtration, the filter-recovered *C. elegans* were immediately poured in TRIzol reagent, stopping all enzymatic activity.

**Fig. 1:**
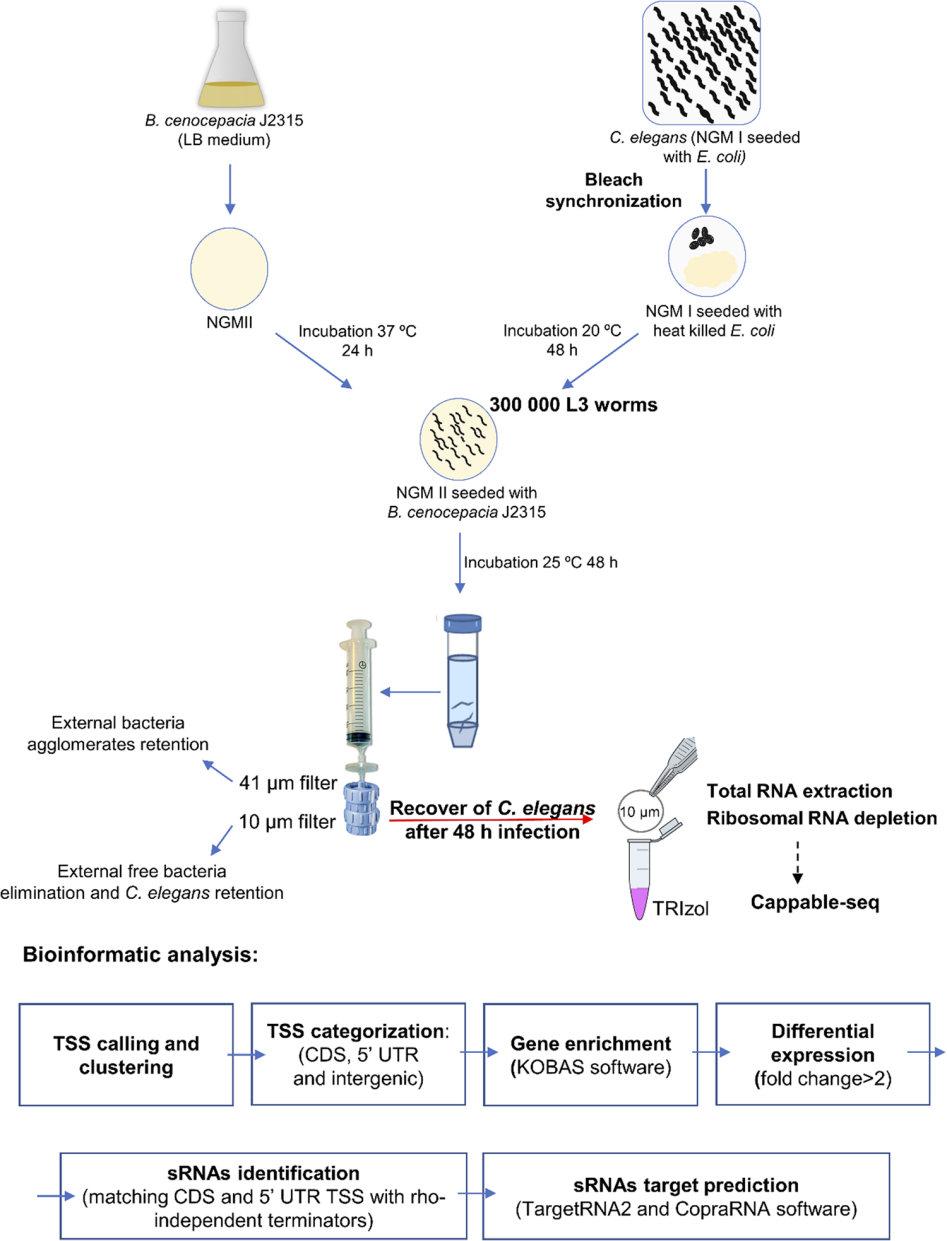
Schematic representation of the methodology implemented to infect *C. elegans* nematodes with *B. cenocepacia* J2315 and recover total RNA to study the bacterial transcriptome in conditions of infection. Abbreviations: CDS, coding sequences; 5′ UTR, 5′ untranslated region

The use of sodium azide and gentamicin, described by others (Zhang et al. 2020), were not used in this work. Instead, the use of the described mechanical procedures guarantees a fast and biocide-free recovering, which minimise alterations on bacterial transcriptome. Despite this, a slightly higher number of bacteria externally attached to the nematodes were collected, but this number is still less than 10% of the total number of bacteria counted inside the *C. elegans*. An illustrative scheme of the protocol developed for the extraction of the *B. cenocepacia* RNA under conditions of infection is shown in Fig. 1. *B. cenocepacia* growing in NGM II plates under the conditions described for the infections assay were also collected to be used as control.

### The transcriptome of *B. cenocepacia* J2315 under infection conditions

#### Determining transcription start sites

After extraction, the total RNA of controls and infection samples was examined by capillary electrophoresis to check the general quality of the samples and the estimated ratio of bacterial/eukaryotic RNA (Supplemental Fig. S1A). The RNA profiles of samples from bacteria infecting the *C. elegans* were distinct from the RNA profiles of bacteria growing in NGMI medium, and like those of *C. elegans* (Supplemental Fig. S1A). These results suggest that RNA samples from infecting bacteria contained a scarce amount of bacterial RNA compared to the RNA of the eukaryotic host. After ribosomal RNA depletion, 5 to 10% of the RNA from both samples and controls were recovered (Supplemental Table S3). This is in agreement with other studies showing that ribosomal RNA accounts for more than 90% of the total RNA in a cell (Sawana et al. 2014). Approximately 0.2% of the total RNA was recovered after the enrichment of the samples in bacterial RNA. This was expected considering the abundance of eukaryotic RNA in samples from the infection process.

**Fig. 2:**
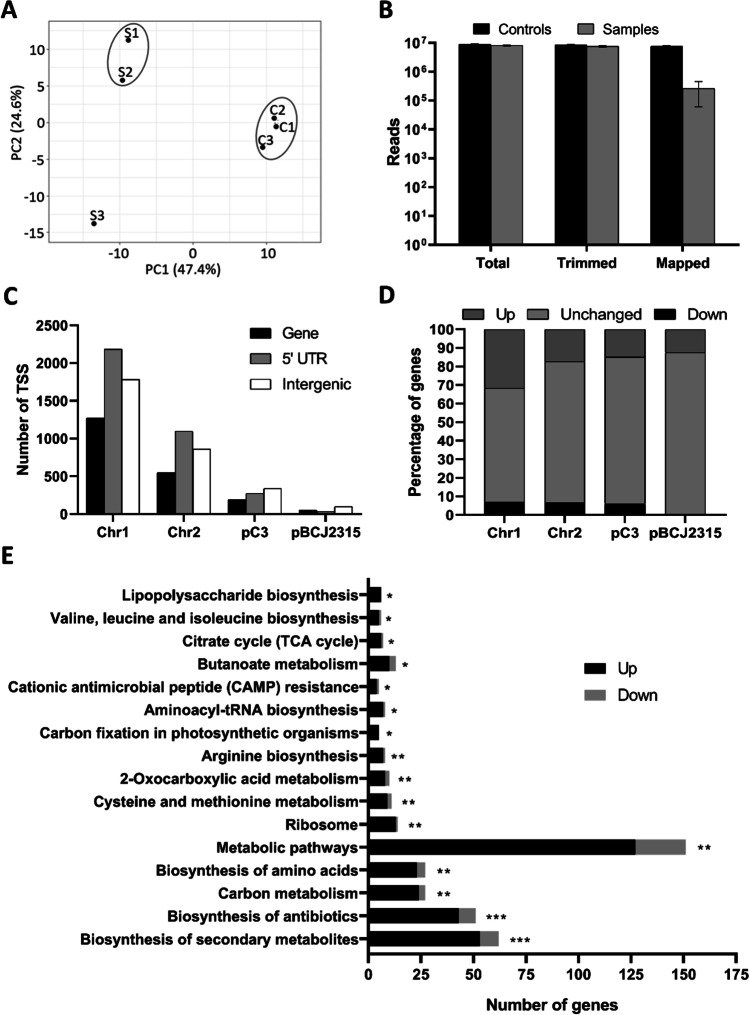
Quality control and characterization of *B. cenocepacia* J2315 Cappable-seq dataset. **A** Principal component analysis (PCA) plots of control and infection samples. **B** Read counting: total, trimmed, and reads mapped to the reference genome of *B. cenocepacia* J2315. **C** Number of TSSs obtained per replicon: gene – TSS located up to 100 nt upstream of a gene, 5′ UTR – TSS located from 100 to 300 nt upstream of a gene, intergenic – TSS located over 300 nts upstream of a gene. **D** Percentage of gene-related TSSs upregulated (twofold), unchanged (any difference in expression level or no expression), and downregulated (twofold) on infection conditions in each replicon. **E** KEGG pathways overrepresented by the altered genes of *B. cenocepacia* J2315 infecting *C. elegans* N2. S1, S2, S3 – *B. cenocepacia* J2315 samples collected from *C. elegans* after 48 h of infection. C1, C2, C3 – *B. cenocepacia* J2315 control samples grown on NGMII agar plates. The Fisher’s exact test was used to determine the statistical significance of the results and the calculated *p*-value is represented by * when *p*-value < 0.05, ** when *p*-value < 0.01, and *** when *p*-value < 0.001

To evaluate the RNA-seq data, a gene expression principal component analysis (PCA) plot was performed and a heatmap was generated, providing insights into the association between all the samples analysed (Fig. 2A and Supplemental Fig. S1B). A clear differentiation is observed between samples from the bacteria infecting *C. elegans* (S samples) and the controls (C samples). All the C samples presented a significant similarity regarding the first two principal components, whilst sample S3 from the infection experiments was less correlated with the samples S1 and S2, probably due to the smaller number of reads. Although about 7 to 9 million raw and trimmed reads were obtained for each control or infection sample (Supplemental Table S4), the number of mapped reads against the *B. cenocepacia* J2315 reference genome decreased significantly in all the infection samples (less than 8% of the trimmed reads), indicating the predominance of eukaryotic RNA in these samples even after the enrichment in bacterial RNA. Figure 2B shows a clear difference between the mapped reads of the controls and infection samples.

Considering the limitations of *B. cenocepacia* J2315 genome annotation to identify the coding and non-coding transcriptome, we initiated data analysis by identifying TSSs. The identification of TSSs was initially performed for controls, since a higher and more reliable number of reads were mapped to the *B. cenocepacia* J2315 reference genome.

A total of 8695 TSSs were identified with the established criteria (Supplemental Table S5). The majority of the TSSs were located in chromosomes 1 and 2 on the putative 5′ UTRs. TSSs located up to 100 nts upstream a coding sequence (gene TSS) were the less abundant (Fig. 2C). The identification of intergenic TSSs is particularly interesting since they can represent important but non-annotated genes, like non-coding RNAs. Curiously, intergenic TSSs represent the majority of the identified TSSs in *B. cenocepacia* J2315 megaplasmid pC3 and plasmid pBCJ2315 (Fig. 2C). We hypothesise that this either might result from a poor annotation of these replicons or might reflect a higher ratio of non-coding sequences in these replicons. Despite the high number of TSSs obtained in this work, most probably resulting from the presence of alternative TSSs, only 2584 annotated genes were identified, which correspond to 35% of all *B. cenocepacia* annotated genes (Supplemental Table S6). Of the 2584 annotated genes identified, 667 genes show multiple TSSs in our dataset. This might result from the presence of genes, namely non-coding RNAs, without a clear terminator or the presence of primary and secondary TSSs. Different transcripts for the same annotated gene have been reported to be expressed under different environmental conditions, probably reflecting the high adaptability of bacteria (Mejía-Almonte et al. 2020). Interestingly, the TSSs identified in our dataset suggest that different transcripts of some *B. cenocepacia* genes are expressed in controls and infection samples. Also of notice is that some genes have a TSS highly expressed in control and downregulated in the infection samples, whilst TSSs scarcely expressed in the control were found upregulated in the infection samples (Supplemental Table S6). These results evidence a selective expression of distinct transcripts under different environments. One such example is the *BCAL1887* gene that codes for the nucleoside diphosphate kinase Ndk: the expression of the TSS located 227 nts upstream of the start codon is increased 16 times in infection samples compared to control samples, whilst the TSS located 40 nts upstream of the start codon is 5 times less expressed in infection samples than in the control samples (Supplemental Table S6).

#### Revealing the coding transcriptome

To unveil genes differently expressed under infection conditions, a comparison of normalised values of gene expression between control and infection samples was performed. Figure 2D shows that the expression levels of most of the annotated genes remained unchanged in bacteria infecting the *C. elegans*, including those not detected in the infection sample dataset. Nevertheless, a considerable percentage of genes presented significant changes in their expression levels. The percentage of dysregulated genes decreased according to their location, respectively, in chromosomes 1 and 2, megaplasmid pC3, and plasmid pBCJ2315. The expression of almost 38% of the genes located in chromosome 1 was significantly altered in infection samples, most of them being upregulated.

After 48 h of *C. elegans* infection, the most significantly affected pathways detected in *B. cenocepacia* were the biosynthesis of secondary metabolites and the biosynthesis of antibiotics (*p*-value < 0.001) (Fig. 2E). These pathways are involved mainly in the production of non-essential molecules for bacterial survival, but are of critical importance for environment interactions (Pandey et al. 2021). In this work, a KOBAS software gene-list enrichment analysis showed that most of the genes identified by Wong et al. (2018) as essential for *B. cenocepacia* growth on NGMI and survival in *C. elegans* also belong to the KEGG pathways related with the biosynthesis of secondary metabolites and biosynthesis of antibiotics (Supplemental Table S7) (Wong et al. 2018). In addition, a significant number of the genes here identified as differently expressed in infection conditions match to those identified by Wong et al. (2018) as essential for *B. cenocepacia* survival in *C. elegans* (Fisher statistic test, *p*-value < 0.05).

Genes previously identified as virulence factors of *B. cenocepacia* were found to be differently expressed in *B. cenocepacia* J2315 during *C. elegans* infection. The majority were upregulated (22 genes out of the 29 differently expressed virulence factors) and some of them are involved in processes related with bacteria sensing and interaction with the surrounding environment, namely two-component systems (e.g. *ompR*), quorum sensing (e.g. *rpfR* and *cepI*), and bacterial secretion systems (e.g. *virB1* and *gspD*) (Supplemental Table S6). The extracytoplasmic function sigma factors *orbS* (*BCAL1688*) and *ecfD* (*BCAL2360*), two major transcriptional regulators in response to environmental changes (de Dios et al. 2021), were found more than 6 times upregulated (Supplemental Table S6). Curiously, the genes that code for the Hfq chaperone (*BCAL1879*) and the acyl carrier protein AcpP (*BCAL0995* and *BCAL2875*), previously described by our research group as essential for Bcc full virulence in *C. elegans* (Sousa et al. 2008, 2010), were found to be upregulated in *B. cenocepacia* J2315 during *C. elegans* infection (Supplemental Table S6). The LysR family protein ShvR, a pleiotropic regulator (Subramoni et al. 2011; Gomes et al. 2018), was also found to be downregulated in *B. cenocepacia* during *C. elegans* infection (Supplemental Table S6).

#### Non-coding RNA identification and characterization

**Fig. 3:**
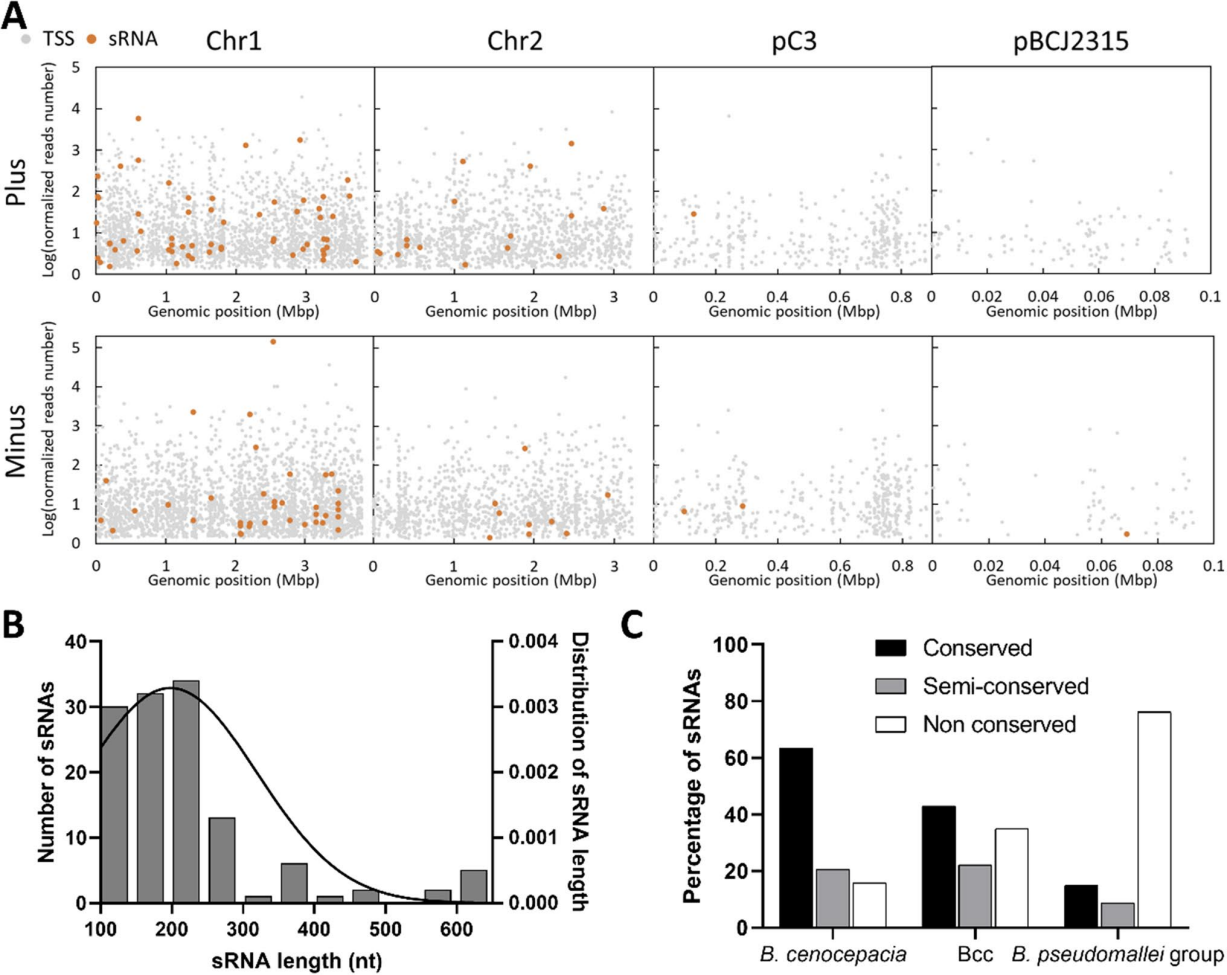
Genomic distribution and characteristics of the *B. cenocepacia* J2315 sRNAs identified in this work. **A** TSS and sRNA distribution by replicon. Plus: positive or sense strand; minus: negative or antisense strand. **B** sRNA length distribution. The average sRNA length is 200 nts, which is represented by the Gaussian distribution. **C** sRNA conservation. The majority of the identified sRNAs are conserved in *B. cenocepacia*, conserved or semi-conserved in Bcc, and non-conserved in *B. pseudomallei* group (classification criteria are detailed in Supplemental Table S9)

To identify the non-coding RNAs expressed by *B. cenocepacia* when infecting *C. elegans*, the identified 5′UTR and intergenic TSSs were matched with the predicted rho-independent terminators, since sRNAs are generally terminated by this type of terminators (Chen et al. 2019). To maximise the number of identified sRNAs, all the TSSs identified in controls (8695) (Supplemental Table S5) and infection samples (186) were considered (Supplemental Table S8). This analysis led us to identify 130 putative sRNAs for 100 rho-independent terminators (Supplemental Table S9). In addition to the 130 putative sRNAs, sRNAs already annotated on NCBI database, were also found as expressed under infection conditions (Supplemental Table S6). A total of 108 putative sRNAs, corresponding to 83 rho-independent terminators, are described here for the first time. The identified sRNAs are mainly located on chromosome 1 (Fig. 3A), with an average length of approximately 200 nucleotides (Fig. 3B). Most of their sequences are conserved amongst *B. cenocepacia* strains, with only 15% not conserved (Fig. 3C; Supplemental Table S9). About 65% of the identified sRNAs were conserved or semi-conserved amongst Bcc members. The number of conserved sRNAs is relatively low in the phylogenetic related group of *Burkholderia pseudomallei*, which can cause melioidosis in humans (Sawana et al. 2014). Of the 168 sRNAs previously described in Bcc (Pita et al. 2018), 31 were found to be expressed in the conditions tested in this work, but only 22 were identified (Supplemental Table S9) using the established sRNA identification methodology, which excluded the NCBI annotated sRNAs. Thirteen sRNAs previously identified in *B. cenocepacia* biofilms were found to be expressed in our dataset, and 7 of them were found to be dysregulated in *B. cenocepacia* during *C. elegans* infection (Sass et al. 2017) (Supplemental Table S9).

The first two columns show the differential gene expression relative to a fold change of the 6 sRNAs selected, measured during *C. elegans* infection relative to controls, and their conservation level. The predicted targets were identified by the locus tag and the virulence factors were highlighted in bold. The function of the predicted targets, their differential expression during *C. elegans* infection, the location of the predicted sRNA binding region, and the statistical significance of the predicted interaction were also included. The *p*-values were predicted for the probability of finding an interaction in random sequences with a score greater than or equal to the score of the observed interaction and were represented by * when the *p*-value < 0.05, ** when the *p*-value < 0.01, *** when the *p*-value < 0.001, and **** when the *p*-value < 0.0001.

^a^Genes that were not directly identified by bioinformatics tools. The binding regions identified as being upstream of the targets predicted and listed on Supplemental Table S10 are in fact located on the genes listed in this table. The targets predicted were BCAL1262, BCAM1946, BCAL2829, BCAL1675, and BCAL3515.

^b^The differential expression mentioned refers to the first gene of the operon from which this gene is transcribed.

### RIT2a, RIT11b, RIT32, RIT43, RIT55, and RIT98 are sRNAs directly affecting *B. cenocepacia* virulence?

Based on the different levels of expression between control and infection samples, degree of conservation amongst Bcc members, and predicted targets, 5 sRNAs were selected for further characterization: RIT11b, RIT32, RIT43, RIT55, and RIT98, that were previously identified by Sass et al. (2017) as ncS06, ncS16, nc5U42, ncS11, and ncRI12, respectively. RIT11b, RIT32, and RIT55 were downregulated in infection samples, with a fold change of − 4, − 27, and − 153, respectively, whereas RIT43 and RIT98 were upregulated, with a fold change of 8 and 59, respectively (Table 2 and Supplemental Table S9). RIT2a, previously identified as ncS01 (Sass et al. 2017), was also selected due to the high expression and high conservation levels amongst Bcc and *B. pseudomallei* group species. Despite the high level of expression, this sRNA was not found as differentially expressed under infection conditions compared to controls. All the 6 selected sRNAs are highly conserved amongst Bcc species and RIT32, RIT55, and RIT98 are also conserved amongst the *B. pseudomallei* group (Table 2 and Supplemental Table S9). TargetRNA2 and CopraRNA were used to predict the targets of the selected sRNAs. Amongst the predicted targets, genes coding for virulence factors, sigma factors, and genes required for Bcc survival in different environments were selected (Table 2). To increase the probability of selecting direct targets of these sRNAs, the location of the predicted RNA-RNA interaction site on the mRNA was also considered in the analysis of the targets that are most likely to bind directly to each sRNA. Amongst the selected sRNAs, RIT11b presented the highest number of predicted targets, nine of them encoding virulence factors.

**Table 2:** Predicted targets for the selected sRNAs RIT2a, RIT11b, RIT32, RIT43, RIT55, and RIT98

		Predicted targets				
*sRNA/DF C. elegans*	Conservation	NCBI locus tag	Function/system	Regulation	TargetRNA2	CopraRNA
*RIT2a (ncS01)* + *1.35x*	Bcc	*BCAM1011*	EPS biosynthesis	-	51|68****	52|67***
		*BCAL1261*^*a*^	*carAB* operon	Up^b^	417|435****	419|443**
		*BCAM2060*	Metal ion transport	Up	-	12|29*
		*BCAM1947*^*a*^	RND-9 efflux pump	-	1096|1106**	-
		*BCAL3233*	Glycosyltransferase	Up	4|16*	-
*RIT11b (ncS06)* * − 3.74x*	Bcc	*BCAL1625*	tRNA processing	Up	51|65****	− 35|37*
		*BCAL3116*	Glycosyltransferase	-	− 1|16****	− 198|− 149*
		*BCAL0481*	PG biosynthesis	-	1|32****	64|100*
		*BCAM2688*	Isomerase	-	− 15|1**	− 66|19*
		*BCAL3351*	Pyrimidine biosynthesis	Up	37|65*	5|100*
		*BCAL0207*	Oxidative stress resp.	-	-	− 38|− 5*
		*BCAL0276*	Type IV pilus	-	− 153|− 139*	-
		*BCAL0337*	Type VI secretion	Up^b^	4|31*	-
		*BCAL2011*	Two-component system	Up	-	43|72*
		*BCAL2830*^*a*^	Two-component system	-	1328|1345****	-
		*BCAL3503*	Flagellar biosynthesis	-	-	32|65*
		*BCAM1867*	Macrophage survival	Up	− 138|− 120**	-
		*BCAM2107*	Oxidative stress resp.	-	− 132|− 118*	-
		*BCAM2169*	Membrane transporter	Down	− 156|− 138*	-
*RIT32 (ncS16)* * − 26.66x*	Bcc *B. pseudomallei*	*BCAS0099*	LacI family regulator	Up	− 68|− 37*	− 66|− 37*
		*BCAM0918*	Sigma factor RpoD	-	-	− 299|− 255*
		*BCAM2057*	Type III secretion	-	-	− 24|− 7**
		*BCAM1946*	RND-9 efflux pump	-	-	− 21|− 2*
		*BCAS0104*	Cell adhesion	-	− 15|− 6*	-
		*BCAL0113*	Cell adhesion	-	− 18|3*	-
		*BCAL0824*	*C. elegans* survival	-	-	− 68|− 4*
		*BCAL3471*	Cell division	Up	-	40|96***
*RIT43 (nc5U42)* + *7.73x*	Bcc	*BCAL2349*	Ribosomal protein S15	-	35|55**	− 111|− 96**
		*BCAL3400*	Pyrimidine biosynthesis	-	− 64|− 49*	-
		*BCAL1995*	AHL, quorum sensing	-	-	0|21***
		*BCAL0739*	Phosphoglyceromutase	-	-	− 55|− 32*
		*BCAL0401*	Pentose-phosphate shunt	-	-	− 60|− 7**
*RIT55 (ncS11)* * − 153.25x*	Bcc *B. pseudomallei*	*BCAL2830*	Two-component system	-	-	47|78*
		*BCAL2211*	Two-component system	-	-	− 153|− 129**
		*BCAL1369*	Sigma factor 70 EcfC	-	-	− 156|− 119*
		*BCAL3152*	Sigma factor	-	-	− 107|− 44**
		*BCAL1674*^*a*^	RND-3 efflux pump	-	-	1059|1108**
		*BCAM2060*	Metal ion transport	-	-	− 104|− 68*
*RIT98 (ncRI12)* + *59.06x*	Bcc *B. pseudomallei*	*BCAS0104*	Cell adhesion	-	− 14|− 5*	-
		*BCAL0959*	Cell adhesion	-	− 18|− 5*	-
		*BCAL3121*	*O*-antigen biosynthesis	-	-	− 90|10**
		*BCAL3152*	Sigma factor	-	-	− 85|− 61*
		*BCAL3516*^*a*^	Type II secretion	-	-	391|484*

**Fig. 4:**
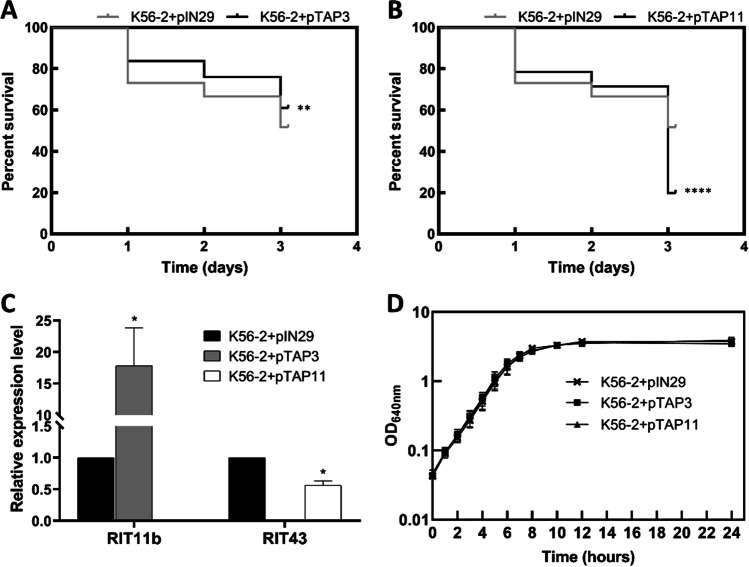
Effect of RIT11b overexpression and RIT43 silencing in *B. cenocepacia* K56-2. **A** and **B** Kaplan–Meier survival plots of *C. elegans* infected with *B. cenocepacia* K56-2 carrying the empty vector pIN29 (control), the pTAP3 plasmid overexpressing RIT11b (**A**), or the pTAP11 plasmid silencing RIT43 using an antisense strategy (**B**). Survival curves show the results of three representative independent assays. Log-rank (Mantel-Cox) test was used to determine significance of survival differences. **C** Relative expression levels of the sRNAs RIT11b and RIT43 quantified by qRT-PCR. Multiple *t* tests (one per row) were used to determine statistical significance. **D** Bacterial growth curves in rich medium (LB), monitored by optical density at 640 nm. C and D graphs show the mean ± SD from a representative of three independent assays. Statistical significance is represented in accordance with *p*-value, *p-value < 0.05, **p-value < 0.01, and ****p-value < 0.0001

To evaluate the direct effect of the selected sRNAs on *B. cenocepacia* virulence towards the *C. elegans* infection model, slow-killing assays were conducted using *B. cenocepacia* K56-2 (a strain very close to *B. cenocepacia* J2315 but genetically easier to handle) carrying pIN29 derivative plasmids overexpressing RIT11b, RIT32, or RIT55, or silencing RIT2a, RIT43, or RIT98. We choose to overexpress RIT11b, RIT32, or RIT55, and silence RIT2a, RIT43, or RIT98, since our results show that these sRNAs were, respectively, downregulated or upregulated in infection conditions compared to controls. Infection assays reveal that the overexpression of RIT11b in *B. cenocepacia* K56-2 attenuated the virulence of this strain in *C. elegans* infection model, whilst the RIT43 silencing had the opposite effect (Fig. 4A, B). In in vitro conditions, both RIT11b overexpression and RIT43 silencing were confirmed by qRT-PCR in *B. cenocepacia* K56-2 carrying the pTAP3 and the pTAP11 plasmids, respectively (Fig. 4C). No effect in *B. cenocepacia* virulence in *C. elegans* infection model was detected when RIT2a or RIT98 was repressed, or when RIT32 or RIT55 was overexpressed (Supplemental Fig. S2). The overexpression of RIT11b or silencing of RIT43 did not affect the growth kinetics of *B. cenocepacia* K56-2, thus indicating that the effects observed in virulence were not due to impaired bacterial growth (Fig. 4D). The attenuated virulence of *B. cenocepacia* observed with RIT11b overexpression is in agreement with our RNA-seq data, where RIT11b was downregulated in *B. cenocepacia* during *C. elegans* infection. Despite the upregulation of RIT43 under infection conditions, an increased virulence of *B. cenocepacia* was observed when RIT43 was silenced by our antisense strategy. This effect may be due to an unknown effect of the selected antisense sequence to silence this sRNA or to a compensatory effect by another regulator.

### RIT11b is a virulence regulator in *B. cenocepacia*

**Fig. 5:**
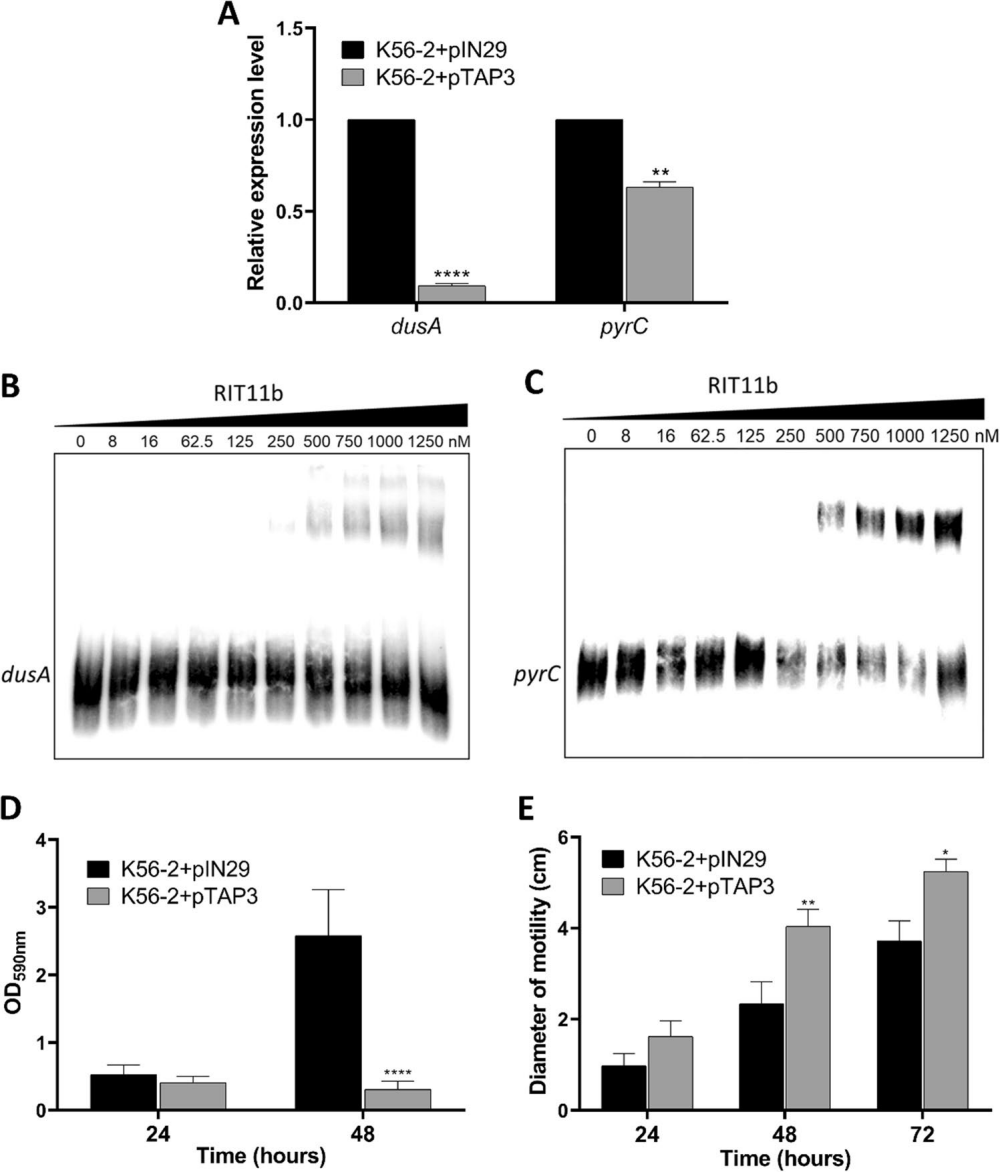
Effect of RIT11b overexpression in *B. cenocepacia* K56-2. **A** Regulatory effect of RIT11b in two predicted targets. Relative expression levels of the mRNAs *dusA* and *pyrC*, when RIT11b is overexpressed in *B. cenocepacia* K56-2 (pTAP3 plasmid). **B** and **C** Electrophoretic mobility shift assay (EMSA). Interactions between the unlabelled sRNA RIT11b and the labelled BCAL1625 (**B**) or BCAL3351 (**C**) RNAs. **D** RIT11b impairs biofilm formation. Comparison of the amount of biofilm formed by *B. cenocepacia* K56-2 with pIN29 or pTAP3 plasmids in polystyrene microtitre plates, after 24 h or 48 h of growth in LB medium. **E** RIT11b increases the *B. cenocepacia* motility. Quantification of the swimming motility diameter of bacterial cells at 24, 48, and 72 h after inoculation onto swimming agar plates. *B. cenocepacia* K56-2 carrying the pIN29 plasmid was used as control. All graphs show the mean ± SD from a representative of three independent assays. Two-way ANOVA was used to assess the statistical significance of biofilm formation and swimming motility, represented in accordance with the *p*-value, *p-value < 0.05, **p-value < 0.01, ***p-value < 0.001, and ****p-value < 0.0001

The role played by RIT11b on the virulence of *B. cenocepacia* K56-2 towards *C. elegans* led us to hypothesise RIT11b as a negative regulator of the expression of genes required for *B. cenocepacia* virulence. Out of the predicted targets for RIT11b (Table 2), *BCAL1625* and *BCAL3351* genes stand out as the RIT11b sole targets identified by the two bioinformatics tools used as being upregulated under infection conditions. *BCAL1625* encodes the tRNA-dihydrouridine synthase A (DusA), whilst *BCAL3351* encodes the dihydroorotase PyrC. The *dusA* and *pyrC* downregulation observed when RIT11b was overexpressed suggests that both *dusA* and *pyrC* mRNAs are regulated by RIT11b (Fig. 5A). Electrophoretic mobility shift assays (EMSA) using increasing concentrations of RIT11b showed that in vitro this sRNA directly interacts with *dusA* and *pyrC* mRNAs (Fig. 5B, C).

In addition to *dusA* and *pyrC*, many other targets were predicted for RIT11b, mostly related to the cell wall and membrane-associated mechanisms, namely peptidoglycan biosynthesis, lipopolysaccharide (LPS) *O*-antigen biosynthesis, epithelial cell adhesion, and biofilm formation, suggesting a role on host colonization and invasion. In vitro assays were performed to determine whether RIT11b would be involved in regulating the motility and biofilm formation of *B. cenocepacia*. The overexpression of RIT11b reduced biofilm formation, especially after 48 h of growth, and promoted swimming motility in *B. cenocepacia* K56-2 (Fig. 5 D and E). Considering all the results obtained, we hypothesise that RIT11b is a pleiotropic regulator in *B. cenocepacia*.

## Discussion

Despite the advances in therapies, infections by *B. cenocepacia* and other Bcc bacteria remain a life-threatening to CF patients due to their resistance to multiple antibiotics, and the unpredictable outcome of infections. Understanding the molecular details and mechanisms underlying the contribution of sRNAs to Bcc bacteria-host interaction is expected to allow the identification of potential interesting targets for the rational development of novel strategies to combat infections caused by Bcc, as well as to predict the outcome of infections caused by these bacteria. Although several studies have identified more than a hundred putative sRNAs encoded within the genome of *B. cenocepacia* J2315, there is an obvious lack of knowledge regarding the non-coding virulome of these bacteria.

A methodology to unveil the *B. cenocepacia* non-coding transcriptome under real infection conditions was developed in this work using *C. elegans* as infection model. Over the past two decades, the nematode *C. elegans* has been used as an infection model to assess the impact of some genes on virulence of Bcc bacteria (Köthe et al. 2003; Hunt et al. 2004; Cardona et al. 2005). Pleiotropic regulators, genes involved in quorum-sensing mechanisms, lipopolysaccharide *O*-antigen biosynthesis, general stress responses and in nutrient acquisition have been shown to attenuate the virulence of these bacteria in *C. elegans* (Köthe et al. 2003; Sousa et al. 2010; Wong et al. 2018). Considering the poor characterization of the *B. cenocepacia* non-coding virulome, this infection model was used to identify sRNAs that are involved in bacterial virulence and to try to establish an association with the predicted targets that are also involved in this process. As the main objective was the identification of sRNAs that control the *B. cenocepacia* virulence, samples collected under infection conditions and controls were sequenced by Cappable-seq, a method that enables a robust TSS determination in bacteria (Ettwiller et al. 2016). This methodology allowed the identification of 8695 TSSs under the tested conditions. Although using distinct methods and conditions, 10843 TSSs were previously identified in *B. cenocepacia* biofilms using a differential RNA-sequencing methodology, which were cut off to 4010 TSSs after filtering on a minimum of 10 read starts (Sass et al. 2015). Similarly, under biofilm growth conditions, 2089 genes (28% of the annotated genes) were found to be transcribed by *B. cenocepacia* (Sass et al. 2015), close to the 2584 genes identified in this work. Thirty-two percent of the 2584 expressed genes were found to be differentially expressed under infection conditions. The highest percentage of differently expressed genes was found in chromosome 1, which contains a higher proportion of coding sequences involved in core functions, such as DNA replication, protein synthesis, energy metabolism, and cell wall biosynthesis (Wong et al. 2016). In this work, genes related with metabolic pathways, amino acid biosynthesis, translation functions, and LPS biosynthesis were also found to be significantly dysregulated in *B. cenocepacia* infecting *C. elegans* (Fig. 2E), suggesting that the infection process requires a fine tuning of core cellular processes and pathways. Previously, Yoder-Himes et al. (2009) found that under CF conditions, *B. cenocepacia* responds primarily by expressing core housekeeping genes mainly encoded on chromosome 1 (Yoder-Himes et al. 2009). Furthermore, Pimenta et al. (2021) also reported that during the early contacts with giant plasma membrane vesicles (GPMVs) derived from live bronchial epithelial cells, general metabolic pathways are significantly dysregulated. These metabolic changes have also been observed in other bacterial pathogens. Alterations in infection-specific gene expression related with transport and metabolism of amino acids and transcription functions were observed in *V. parahaemolyticus* during all the infection process of *C. elegans* (12–48 h). In addition, genes enriched in translation functions, membrane biogenesis, and energy production were significantly induced at the late stages of infection (Zhang et al. 2020). Still, the most significantly altered KEGG pathway was the biosynthesis of secondary metabolites. These molecules can be antibiotics, mycotoxins, plant growth factors, fungal elicitors, plant and animal host defensins, and quorum-sensing molecules, playing a crucial role in the interactions of bacteria with other surrounding organisms (Martín et al. 2005).

Most of the virulence factors expressed under infection conditions were found to be upregulated during *C. elegans* infection and are mostly related to processes regarding bacteria sensing and interaction with the surrounding environment. Two of the those genes were the extracytoplasmic function sigma factors *orbS* and *ecfD*, which in *Burkholderia* species are activated by iron limitation conditions, managing the production and secretion of siderophores, which chelate iron and facilitate its cellular uptake, constituting an important mechanism for bacterial virulence (Grove 2022). The genes that code for the Hfq chaperone and the LysR family protein ShvR, two pleiotropic regulators, were found to be dysregulated in *B. cenocepacia* J2315 during *C. elegans* infection (Supplemental Table S6). Hfq is involved in the regulation of virulence traits related to secretion systems, alternative sigma factors, outer membrane proteins, polysaccharides, and iron metabolism, and impacts the pathogenicity of several bacterial pathogens, including Bcc bacteria (Sousa et al. 2010; Feliciano et al. 2016). The increased expression of Hfq chaperone under infection conditions strongly suggests that the infection process is post-transcriptionally regulated through small RNAs. ShvR influences the expression of genes for quorum sensing, protease, type II secretion system, and *afc* genes. In *B. cenocepacia*, this regulator is important for biofilm formation, inflammation in a rat lung infection model, and is required for the induction of fatal proinflammatory responses in zebrafish larvae (Subramoni et al. 2011; Gomes et al. 2018). These results highlight the importance of the expression of some virulence factors for the adaptation and successful infections caused by *B. cenocepacia*.

Regarding the non-coding transcriptome, the data analysis focused only on the TSSs identified in the intergenic regions and on the presence of Rho terminators in the vicinity of these TSSs, limiting the number of sRNAs obtained. Of the sRNAs identified by the methodology developed, 108 were described here for the first time, as well as 22 previously described, thus validating our approach. This is a major contribution to the identification of the *B. cenocepacia* non-coding genome, especially to find sRNAs differently expressed under infection conditions. Amongst the already described sRNAs, some sRNAs previously identified in *B. cenocepacia* biofilms were found to be dysregulated in *B. cenocepacia* during *C. elegans* infection, like the ncS35 sRNA. The ncS35 sRNA was described as a sRNA that can reduce the metabolic rate and growth, possibly to protect *B. cenocepacia* against stressors, and increase the bacterial survival under unfavourable conditions, like the ones found in host–pathogen interactions (Kiekens et al. 2018). The upregulation of this sRNA in *B. cenocepacia* during *C. elegans* infection may be important to finely tune the expression genes involved in metabolic pathways that are determinant for *B. cenocepacia* adaptation to host environment.

To understand whether the identified sRNAs directly affected the virulence of *B. cenocepacia* against *C. elegans*, six sRNAs were selected for further characterization based on their differential expression in *C. elegans* infection, high conservation amongst Bcc strains, and their predicted targets. Regarding the predicted targets, those coding for virulence factors, sigma factors, and genes required for Bcc survival in different environments were preferentially selected. Although several genes involved in *B. cenocepacia* virulence are predicted targets of the RIT2a, RIT32, RIT55, and RIT98 sRNAs, under the tested conditions it was not possible to establish a direct effect of the expression of these sRNAs and *B. cenocepacia* virulence. However, the sRNA RIT11b, which was found as downregulated under infection conditions, has proved to be involved in *B. cenocepacia* virulence. RIT11b was overexpressed in *B. cenocepacia* K56-2 and attenuated the bacterium virulence in *C. elegans*, highlighting the active role of this sRNA in virulence.

Virulence factors involved in stress responses, secretion systems, motility, and macrophage survival were predicted targets of RIT11b, and some of them (*BCAL2011*, *BCAM1867*, *BCAL0337*, *BCAL1625*, and *BCAL3351*) were found to be upregulated in *B. cenocepacia* during *C. elegans* infection. In addition to be upregulated, *dusA* (*BCAL1625*) and *pyrC* (*BCAL3351*) have also been described to be involved in bacterial virulence, and we showed in vitro the direct binding of RIT11b with the mRNA of these two genes. qRT-PCR assays revealed that *dusA* and *pyrC* can be negatively regulated by RIT11b. PyrC, a key player of pyrimidine nucleobase biosynthetic process, was also found to be overexpressed in *P. aeruginosa* during chronic infections (Winsor et al. 2008; Naughton et al. 2011), suggesting that the *C. elegans* infection process by *B. cenocepacia* resembles a chronic infection. Regarding DusA, this protein was previously identified as essential for *B. cenocepacia* survival in *C. elegans* (Wong et al. 2018). The *dus* genes family have been found to be downregulated during heat shock stress in *Clostridium botulinum*, in *E. coli* upon cell death mediated by bactericidal antibiotics, or in *Bdellovibrio bacteriovorus* predation (Kohanski et al. 2007; Lambert et al. 2010; Liang et al. 2013). The *dusB* deletion mutant was also associated with a reduced biofilm production by *P. aeruginosa* and *Actinobacillus pleuropneumoniae* (Müsken et al. 2010; Grasteau et al. 2011). Interestingly, *dusA* deletion significantly reduced *Neisseria meningitidis* adherence to human epithelial cells (Hey et al. 2013). Bacterial adherence is also fundamental for host colonization by *B. cenocepacia* and the bacterium pathogenicity (Leitão et al. 2010; Kumar et al. 2020). Therefore, we hypothesise that DusA plays a role in *B. cenocepacia* similar to the *N. meningitidis* homologue.

Many other targets were also predicted for RIT11b, mostly related to the cell wall and membrane-associated mechanisms, namely peptidoglycan biosynthesis, lipopolysaccharide (LPS) *O*-antigen biosynthesis, epithelial cell adhesion, and biofilm formation, suggesting a pleiotropic role of this sRNA on host colonization and invasion. When overexpressed in *B. cenocepacia* K56-2, RIT11b reduced biofilm formation and promoted swimming motility. This negative correlation between the biofilm formation and swimming motility was also recently described by Sass et al. (2022) when increasing the expression of *wspH* in *B. cenocepacia* (Sass et al. 2022)*.* The overexpression of *wspH*, a two-component hybrid system kinase-response regulator protein, led to accelerated pellicle biofilm formation and reduced swimming motility. This kinase had previously been described as a biofilm regulator and was linked to the formation of small colony variants (SCVs) (Cooper et al. 2014). Curiously, when we predicted the interaction between *wspH* mRNA and RIT11b sRNA using IntaRNA, multiple-interaction sites were predicted (energy score of the predicted interactions: − 14 to − 6.6 kcal/mol). The impaired biofilm formation and the increased swimming motility observed when RIT11b was overexpressed in *B. cenocepacia* J2315 might result from a downregulation of *wspH* by RIT11b.

Several other sRNAs have been described as pleiotropic modulators of bacterial virulence, such as *P. aeruginosa* RsmZ, ReaL, ErsA, and SrbA (Liu et al. 2022); *Staphylococcus aureus* RsaA and Teg49 (Menard et al. 2022); and *E. coli* and *Salmonella enterica* McaS, OmrA, and OxyS (Mitra and Mukhopadhyay 2023). Therefore, whilst most of these sRNAs positively modulate bacterial virulence and biofilm formation, RIT11b was downregulated when *B. cenocepacia* was infecting *C. elegans* and its overexpression reduced bacterial virulence and biofilm formation. OmrA and RydC, in Gram-negative bacteria, and AscicR and csRNA1-1, in Gram-positive bacteria, are some of the few sRNAs that also negatively modulate biofilm formation (Mitra and Mukhopadhyay 2023).

Knowledge on the regulatory roles of sRNAs is crucial to understand mechanisms underlying bacterial infection and host–pathogen interaction processes, and to find novel ways to control infections. A closer look to less obvious targets, like *dusA* and *pyrC*, is also required due to their importance for bacteria virulence. Beyond *dusA* and *pyrC*, multiple and diverse targets were predicted for RIT11b, from quorum-sensing to pyrimidine metabolism. Also, in addition to the role played by RIT11b on *B. cenocepacia* virulence, changes in biofilm formation ability and motility were also observed when RIT11b was overexpressed, suggesting that other targets should be regulated by this sRNA. We therefore hypothesise that RIT11b is a pleiotropic regulator impacting the ability of *B. cenocepacia* to infect a host, being the first characterization of a sRNAs directly involved in *B. cenocepacia* virulence.

## Supplementary Information

Below is the link to the electronic supplementary material.

Supplementary file1 (PDF 242 KB)

Supplementary file2 (XLSX 2116 KB)

## Data Availability

All data generated or analysed during this study are included in this published article, as supplementary information files. Cappable-seq data is available under Array Express accession number E-MTAB-12592.
