# Supplementary material for: Identification of Burkholderia cenocepacia non-coding RNAs expressed during Caenorhabditis elegans infection
Source: Appl Microbiol Biotechnol. 2023 Apr 25;107(11):3653–71. doi: 10.1007/s00253-023-12530-3 (PMC10175445; doi:10.1007/s00253-023-12530-3)
Supplement: Supplementary file 1 — Supplementary file1 (PDF 242 KB) [file 253_2023_12530_MOESM1_ESM.pdf]

Supplemental Material

Identification of *Burkholderia cenocepacia* non-coding RNAs expressed during *Caenorhabditis elegans* infection

Applied Microbiology and Biotechnology

Tiago Pita [a, b, c], Joana R. Feliciano [a, b, c*], Jorge H. Leitão [a, b, c *]

[a] iBB-Institute for Bioengineering and Biosciences, Instituto Superior Técnico, Universidade de Lisboa, 1049-001 Lisbon, Portugal

[b] Department of Bioengineering, Instituto Superior Técnico, Universidade de Lisboa, 1049-001 Lisbon, Portugal

[c] Associate Laboratory i4HB-Institute for Health and Bioeconomy at Instituto Superior Técnico, Universidade de Lisboa, Av. Rovisco Pais, 1049-001 Lisbon, Portugal

* Correspondence (J.H.L): e-mail: jorgeleitao@tecnico.ulisboa.pt, telephone number: +35 21 8417688, (J.R.F.): email: joana.feliciano@tecnico.ulisboa.pt, telephone number: +35 21 8417688

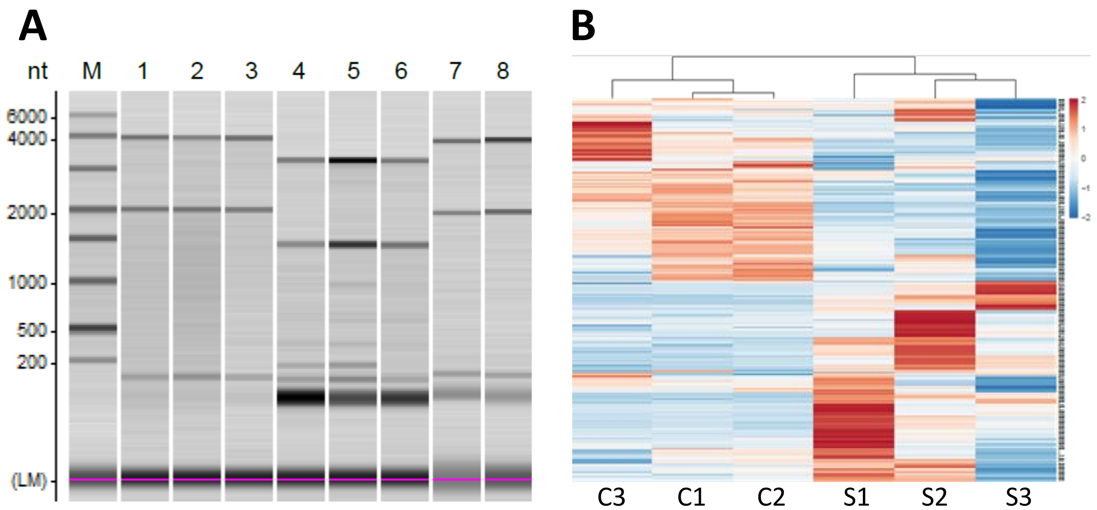

Fig. S1 Quality of total RNA and visualization of CappableSeq results. (A) Capillary electrophoresis of total RNA extracted from *C. elegans* infected with *B. cenocepacia* J2315 (lanes 1, 2, 3), bacterial controls (lanes 4, 5, 6) and host controls (lanes 7, 8). (B) Heatmap, obtained from ClustVis web tool. S1, S2, S3 – *B. cenocepacia* J2315 samples collected from *C. elegans* after 48h of infection. C1, C2, C3 - *B. cenocepacia* J2315 control samples grown on NGMII agar plates.

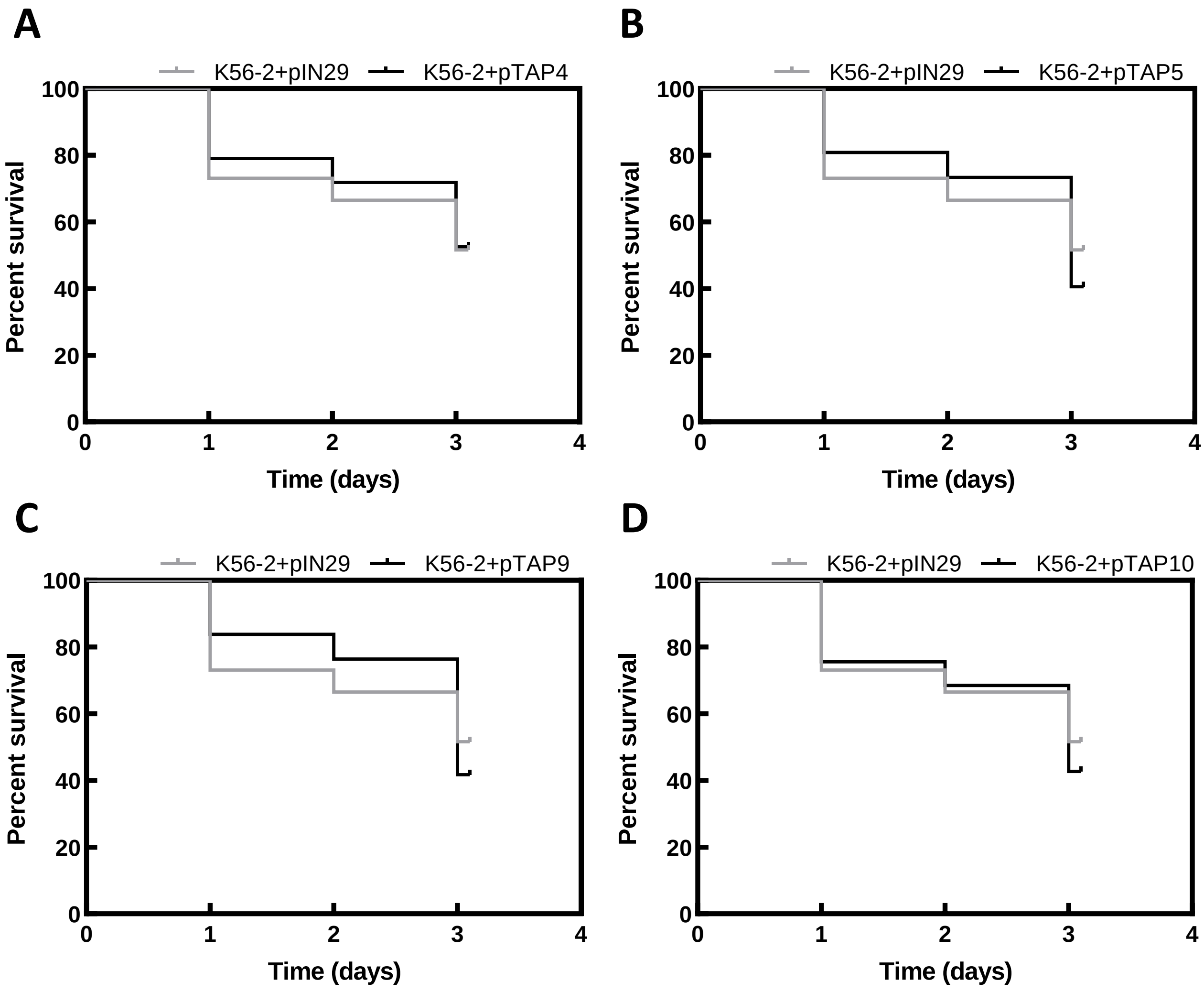

Fig. S2 Kaplan-Meier survival curves of *C. elegans* infected with *B. cenocepacia* K56-2 carrying the empty vector pIN29 or this vector overexpressing or silencing specific sRNAs. (A) RIT32 overexpression, (B) RIT55 overexpression, (C) antisense sequence for RIT2a silencing, (D) antisense sequence for RIT98 silencing. Graph shows three representative independent assays. No significant alterations were found using the log-rank (Mantel-Cox) test.
